# Speed and quality of interbody fusion in porous bioceramic Al_2_O_3_ and polyetheretherketone cages for anterior cervical discectomy and fusion: a comparative study

**DOI:** 10.1186/s13018-023-03625-8

**Published:** 2023-03-03

**Authors:** Roman Kostysyn, Pavel Ryska, Jiri Jandura, Iva Selke-Krulichova, Pavel Poczos, Tomas Hosszu, Tomas Cesak

**Affiliations:** 1 Department of Neurosurgery, Faculty of Medicine in Hradec Kralove, University Hospital Hradec Kralove, Charles University, Hradec Kralove, Czech Republic; 2 Department of Radiology, Faculty of Medicine in Hradec Kralove, University Hospital Hradec Kralove, Charles University, Hradec Kralove, Czech Republic; 3 Department of Medical Biophysics, Faculty of Medicine in Hradec Kralove, Charles University, Hradec Kralove, Czech Republic

**Keywords:** Quality of fusion, Fusion rate, Subsidence, Non-union, PEEK, Al_2_O_3_

## Abstract

**Background:**

The objective of this prospective randomized monocentric study is to compare the speed and quality of interbody fusion of implanted porous Al_2_O_3_ (aluminium oxide) cages with PEEK (polyetheretherketone) cages in ACDF (anterior cervical discectomy and fusion).

**Materials and methods:**

A total of 111 patients were enrolled in the study, which was carried out between 2015 and 2021. The 18-month follow-up (FU) was completed in 68 patients with an Al_2_O_3_ cage and 35 patients with a PEEK cage in one-level ACDF. Initially, the first evidence (initialization) of fusion was evaluated on computed tomography. Subsequently, interbody fusion was evaluated according to the fusion quality scale, fusion rate and incidence of subsidence.

**Results:**

Signs of incipient fusion at 3 months were detected in 22% of cases with the Al_2_O_3_ cage and 37.1% with the PEEK cage. At 12-month FU, the fusion rate was 88.2% for Al_2_O_3_ and 97.1% for PEEK cages, and at the final FU at 18 months, 92.6% and 100%, respectively. The incidence of subsidence was observed to be 11.8% and 22.9% of cases with Al_2_O_3_ and PEEK cages, respectively.

**Conclusions:**

Porous Al_2_O_3_ cages demonstrated a lower speed and quality of fusion in comparison with PEEK cages. However, the fusion rate of Al_2_O_3_ cages was within the range of published results for various cages. The incidence of subsidence of Al_2_O_3_ cages was lower compared to published results. We consider the porous Al_2_O_3_ cage as safe for a stand-alone disc replacement in ACDF.

## Background

Anterior cervical discectomy and fusion (ACDF) is one of the most frequently indicated surgical procedures for degenerative cervical spine disorders. The main surgical goal of this procedure is to provide decompression of the nervous structures, from which patients benefit the most. The secondary goal is to ensure stability of the spine through a quality interbody fusion and to prevent the onset or progression of kyphosis. To provide fusion, bone grafts or different intervertebral devices for cervical disc replacement are used. Cervical discectomy with bone grafting has long been regarded as the gold standard [1]. The main disadvantage of using bone autograft is the potential post-operative donor-site pain [2–4]. A well-documented disadvantage of bone grafts is the significant risk of bone resorption and reduction of intervertebral space [5, 6]. In general, implantation of tricortical bone grafts requires additional fixation, usually by using an anterior plate [1, 7, 8]. Cages made of non-bioactive materials are widely used, their bioactivity being created by addition of bioactive material or bone. The most widely used cage materials are titanium and polyetheretherketone (PEEK), or their combinations. Implants made of bioactive materials (e.g. hydroxyapatite, bioactive glass, calcium phosphates, aluminium oxide, etc.) are relatively newer. When efficacy evaluation studies are performed, new implants are very often compared to PEEK or titanium cages [9–11].

Aluminium oxide (Al_2_O_3_) is a nontoxic, water-insoluble, very hard and chemically inert material. Bioceramic porous Al_2_O_3_ cervical interbody cages have biomechanical properties similar to those of composite bone [12]. Excellent osteoinductive and osteoconductive properties are attributed to them [13].

There is a lack of publications offering experiences and results with the use of Al_2_O_3_ cages in ACDF. Therefore, it was deemed reasonable to design the present study as a comparison of Al_2_O_3_ cages to the more frequently-used PEEK ones.

## Methods

**Fig. 1:**
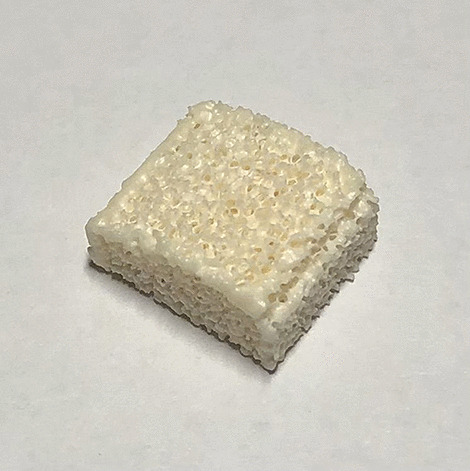
Porous Al_2_O_3_ cage

**Fig. 2:**
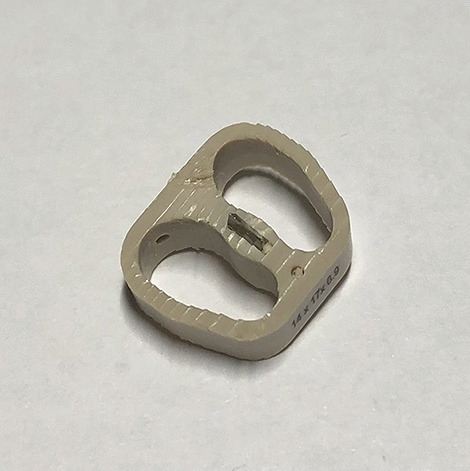
PEEK cage

This prospective, controlled observational, monocentric study was conducted between 2015 and 2021. We hypothesized that a porous bioceramic Al_2_O_3_ cage (Fig. 1) has comparable speed and quality of fusion as a PEEK cage (Fig. 2) with bioactive composite void filler of calcium hydrogen phosphate (CaHPO_4_) and bovine collagen type I. Furthermore, the Al_2_O_3_ cage was expected to create better stability because of a wider contact surface with the vertebral endplates.

All adult patients with symptomatic cervical disc degeneration scheduled for elective one-level ACDF between October 2015 and November 2019 were selected for study enrolment. The inclusion criteria for the study were the following: (1) indication for one-level ACDF predominantly; (2) neurological cervicobrachial symptoms correlated with graphical findings; (3) failure of conservative treatment. Patients were not enrolled or were excluded if any of the following exclusion criteria were met: (1) signs of spinal instability at the indicated level; (2) local inflammation or spondyloarthritis; (3) previous injury of the cervical spine; (4) previous ACDF surgery at any other level; (5) non-availability for FU or inability to complete assessment.

**Fig. 3:**
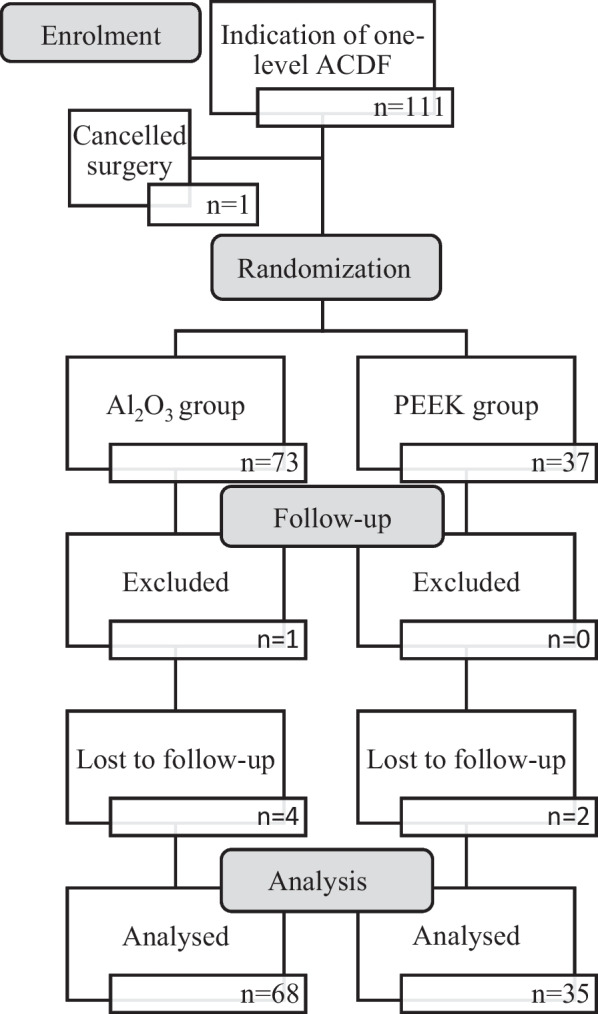
Flow diagram of the study

Initially, 111 patients were included to this study. The unequal allocation randomization method was used for this study. Patients were randomly assigned to one of two groups: ACDF with an Al_2_O_3_ cage or ACDF with a PEEK cage, in a 2:1 ratio. The operations were performed at our department by five experienced surgeons presenting similar skills. The FU of both groups consisted of re-examination of the patients by computed tomography (CT) on the day before surgery, and at designated intervals of 3, 6, 12 and 18 months post-operatively. One patient was excluded from the Al_2_O_3_ group due to late surgical-site infection. Four patients from the Al_2_O_3_ group and two patients from the PEEK group were lost in follow-up. Data of 103 patients were completed and analysed, with 68 and 35 subjects in Al_2_O_3_ and PEEK groups, respectively (Fig. 3).

### Radiological examination and assessment

All cages during the ACDF procedure were implanted under fluoro X-ray control. The day after surgery, plain radiography of the cervical spine was performed in the lateral and anterior–posterior projection to evaluate the correct position of the cage. There are several measuring methods for evaluation of bony fusion achievement (CT, X-plain, X-flexion/extension), with no existing gold standard. The authors of the present study do not consider X-plain imaging as sufficient for final evaluation of bony fusion, despite this imaging being common in our department.

**Fig. 4:**
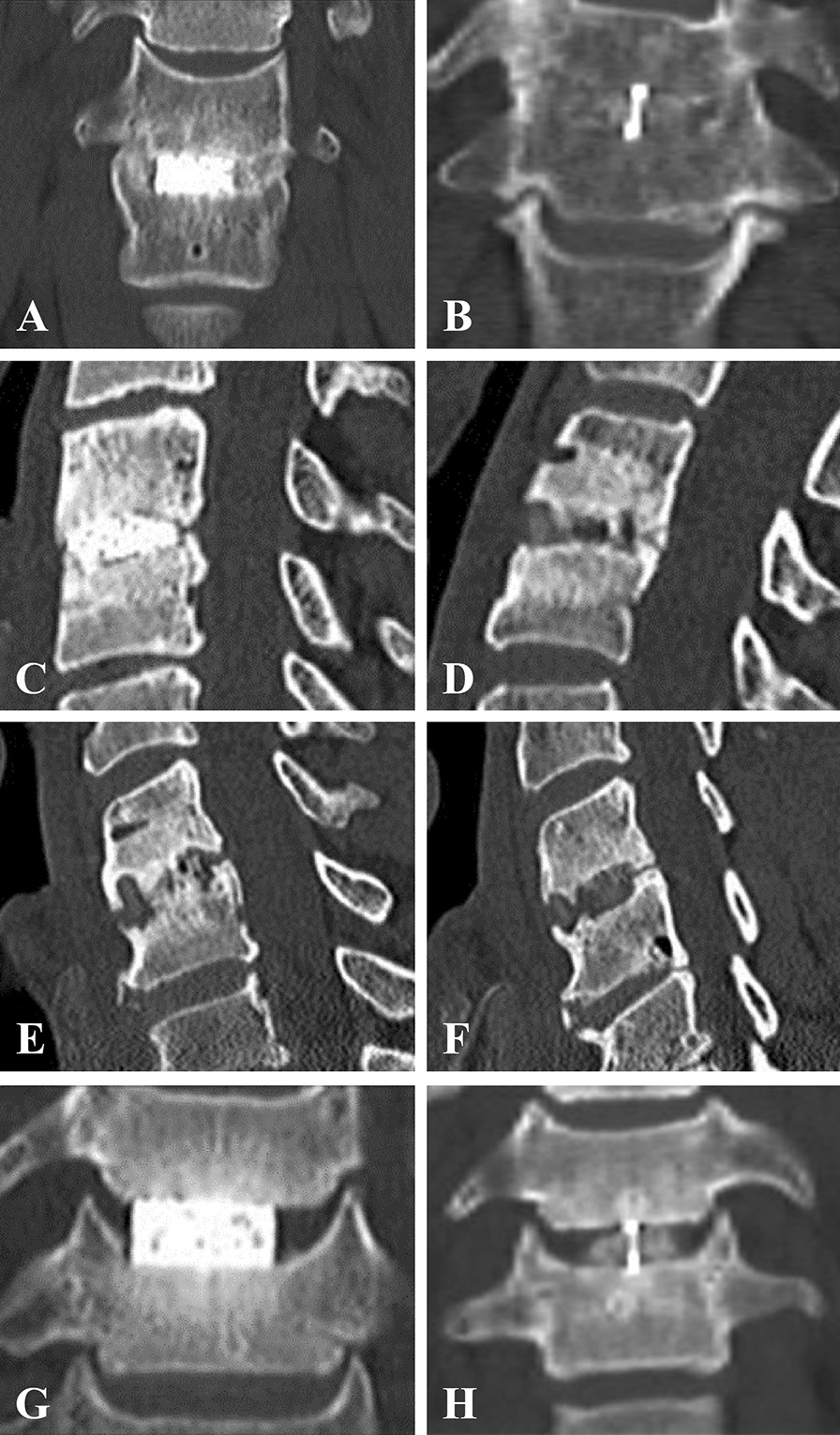
Fusion quality grades on CT scans. Grade I—complete fusion: Al_2_O_3_ cage (**A**), PEEK cage (**B**). Grade II—incomplete bipolar fusion: Al_2_O_3_ cage (**C**), PEEK cage (**D**). Grade III—unipolar fusion: PEEK cage, with trabecular bridging on the left side (**E**), no bridging on the right side (**F**). Grade IV—no signs of fusion: Al_2_O_3_ cage (**G**), PEEK cage (**H**)

CT examinations were performed on Somatom Definition AS + scanner (Siemens, Erlangen, Germany). The standard protocol includes 2 mm scans in axial, coronal and sagittal planes in the osteo-kernel. Evaluation of radiological data was performed by an independent radiologist (PR)*.* Evaluation of initial CT scans included the following parameters: (1) correct position of the cage; (2) first evidence of fusion; and (3) presence of subsidence or osteolysis. The first evidence of bony fusion was defined as the sign of new bone tissue formation in the area of a cage, or incipient trabecular bridging. Subsequently, fusion was assessed and graded qualitatively according to a modified fusion quality scale (Table 1) [14–16]. The presence of any trabecular bridging between the vertebral body and cage or between two endplates confirmed the fusion (grades I, II and III). The fusion was evaluated on four main CT scan views: (1) left coronal, (2) right coronal, (3) left sagittal and (4) right sagittal. The transverse plane was used additionally in cases of doubt. Examples of CT findings of varying fusion quality grades are shown in Fig. 4.

**Table 1:** Fusion quality scale

Grade	Definition	CT imaging (Fig. 4)
I—complete fusion	Bone bridging is visible bilaterally on CT scans in the coronal plane, eventually ventrally or dorsally of a cage in the sagittal plane	A, B
II—incomplete bipolar fusion	Fusion in the area of a cage is presented bilaterally, but it is not completed. Eventually, radiological signs of bone consolidation between the vertebra endplate and the surface of a cage are presented bipolarly	C, D
III—unipolar fusion	Fusion is presented, but only unilaterally or ventrally or dorsally, not all-around the cage	E, F
IV—no fusion (non-union)	No signs of fusion in the area of the cage observed	G, H

### Statistical analysis

All statistical evaluations were performed using NCSS 10 Statistical Software (2015, NCSS, LLC. Kaysville, Utah, USA) and R 4.1.0 (R Core Team. R: A Language and Environment for Statistical Computing. R Foundation for Statistical Computing; 2021). The patients’ age in each group was compared using the Mann–Whitney test. Fisher's exact test was used for analysis of further data: (1) the difference between the sex of patients in both groups, (2) the difference between the occurrence of subsidence and osteolysis in both groups. Extended Fisher's exact test was used for analysis of further intergroup differences: (1) in the surgery level, (2) in the time of fusion initialization, (3) in the distribution of fusion quality grades at 18 months after the surgery, (4) in the time of quality grade I fusion achievement. *P* values < 0.05 were considered significant. The fusion rate for each group was calculated as a percentage of cases with fusion grade III or better.

## Results

### Study population

The demographic and surgical characteristics of Al_2_O_3_ and PEEK groups are shown in Table 2. The difference in patients’ age and sex between both groups was not statistically significant (*p* = 0.707 and *p* = 0.149, respectively).

**Table 2:** Patient population

Study group	Al_2_O_3_	PEEK	*p* value
	(*N* = 68)	(*N* = 35)	
Age in years, median (range)	46 (26–79)	49 (29–73)	0.707
*Sex, n (%)*			0.149
Male	36 (52.9%)	13 (37.1%)	
Female	32 (47.1%)	22 (62.9%)	
*Surgery level, n (%)*			0.700
C4–C5	12 (17.5%)	3 (8.5%)	
C5–C6	34 (50.0%)	22 (63.0%)	
C6–C7	21 (31.0%)	10 (28.5%)	
C7–Th1	1 (1.5%)	0.0 (0%)	

### Initialization and speed of fusion

**Fig. 5:**
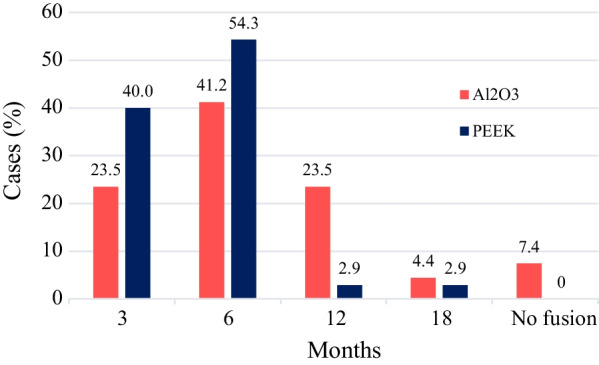
Time of the first evidence of fusion

First evidence of fusion at the 3-month FU was observed in 23.5% of cases in the Al_2_O_3_ group and 40% of cases in the PEEK group (Fig. 5). Within 6 months, signs of fusion initialization were present in 64.7% of cases (sum of cases in 3rd and 6th months) of the Al_2_O_3_ group versus 94.3% in the PEEK group. Even after 12 months (88.2% in sum), the Al_2_O_3_ group had not achieved results as good as the PEEK group after 6 months (94.3%) (Fig. 5). The intergroup difference in fusion initialization was statistically significant (*p* = 0.011).

### Fusion quality and fusion rate

**Fig. 6:**
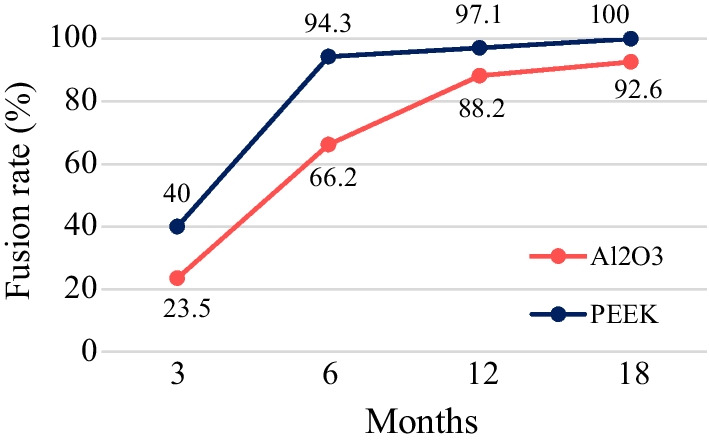
Fusion rate over time

Table 3 contains the distribution of evaluated grades of fusion quality according to the aforementioned scale. At the final FU at 18 months, the presence of different quality fusion was observed in all cases with the PEEK cage and did not occur at all (grade IV) in 5 patients with the Al_2_O_3_ cage (7.4%). The difference in distribution of fusion quality grades between the groups was statistically significant (*p* < 0.001). Satisfactory clinical outcome of patients with non-union did not require further investigation (e.g. X-flexion/extension) or revision surgery. The fusion rate for the Al_2_O_3_ group was 92.6% and PEEK cages showed 100% fusion rate at 18 months. Achievement of fusion in the Al_2_O_3_ group was clearly slower at all time intervals (Fig. 6).

**Table 3:** Fusion quality grade evaluation

Study group	Al_2_O_3_ (*N* = 68)				PEEK (*N* = 35)			
Grade	I	II	III	IV	I	II	III	IV
FU (months)	*n* (%)				*n* (%)			
3	2 (2.9%)	4 (5.9%)	10 (14.7%)	52 (76.5%)	1 (2.9%)	5 (14.3%)	8 (22.8%)	21 (60.0%)
6	5 (7.4%)	8 (11.8%)	32 (47.0%)	23 (33.8%)	5 (14.3%)	15 (42.9%)	13 (37.1%)	2 (5.7%)
12	11 (16.2%)	27 (39.7%)	22 (32.4%)	8 (11.8%)	18 (51.4%)	11 (31.4%)	5 (14.3%)	1(2.9%)
18	26 (38.2%)	24 (35.3%)	13 (19.1%)	5 (7.4%)	27 (77.1%)	3 (8.6%)	5 (14.3%)	0 (0.0%)

### Time to achievement of grade I fusion

**Fig. 7:**
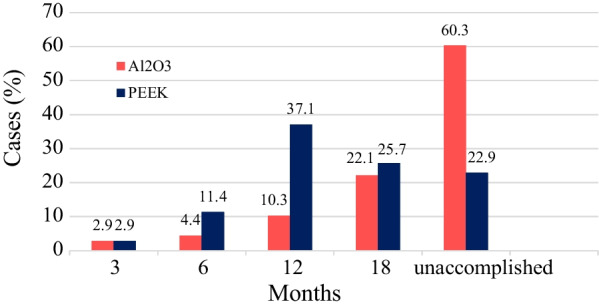
Time to grade I fusion achievement

After the first 3 months, both cages showed the same results (2.9%) for achievement of grade I fusion (Fig. 7). However, between the 3rd and 18th months Al_2_O_3_ cages showed considerably later achievement of grade I fusion than PEEK cages. At the final FU, grade I fusion was observed in 39.7% and 77.1% of cases in the Al_2_O_3_ and the PEEK group, respectively (Fig. 7). This difference was statistically significant (*p* < 0.001).

### Subsidence and peri-implant osteolysis

Subsidence was observed in 22.9% of cases in the PEEK group, which is practically double the 11.8% observed in the Al_2_O_3_ group (Table 4). However, this difference was not statistically significant (*p* = 0.159). Later fusion initialization and later grade I fusion achievement were observed in most cases of subsidence.

**Table 4:** Subsidence and osteolysis [count (%) of cases]

Study group	Al_2_O_3_ (*N* = 68)	PEEK (*N* = 35)	*p* value
	*n* (%)	*n* (%)	
Subsidence	8 (11.8%)	8 (22.9%)	0.159
Osteolysis	3 (4.4%)	5 (14.3%)	0.117
Subsidence in cases of osteolysis	None	3 out of 5	

Osteolysis at the caudal vertebral body endplate was observed in 4.4% and 14.3% of cases in Al_2_O_3_ and PEEK groups, respectively. This intergroup difference was not significant though (*p* = 0,117). A co-occurrence of subsidence and osteolysis can be observed in this study only in PEEK cages—in 3 out of 5 cases, which represents a small series for statistical analysis.

## Discussion

The main focus of our research was the fusion of the Al_2_O_3_ cage in ACDF. We expected that the consolidation of Al_2_O_3_ cages with vertebral body endplates begins between 3 and 6 months, as stated by the manufacturer. However, it was observed only in 64.7% of cases.

In the present study, the fusion rate of 88.2% in Al_2_O_3_ cages at 12 months corresponds with the findings of Noordhoek et al. (an average of 87.6% in different cages). The same authors claim that bony fusion rates do not differ significantly between 12 and 24 months after ACDF and that this is not clinically relevant. Therefore, 12 months FU is sufficient [17]. The fusion rate of Al_2_O_3_ cages at 18 months in our work was 92.6%. This is within the range of published results for various cages in ACDF procedures [1, 9, 17–21].

Finiels reported in 2004 an average fusion rate of 95% at 6 months and 100% at one year after ACDF with Al_2_O_3_ cage. However, that study cohort (61 patients) was diverse—one-level and two-level ACDF, with or without anterior plating [13]. Mostofi et al. published in 2018 preliminary results of using a porous Al_2_O_3_ cage for ACDF in a cohort of 118 patients. Bony fusion evaluated only by plain lateral radiograph was determined in 90.67% at 6 months and in 94.92% at one year after surgery [12]. Both studies showed a higher fusion rate at 6 and 12 months than our findings (66.2% and 88.2%). Our study used CT, which offered us the possibility of more accurate evaluation of the fusion compared to plain radiograph. We are not aware of a study in which CT was used to assess fusion of Al_2_O_3_ cages.

According to CT scans in our study, the fusion starts mainly inside the PEEK cages, where there are two bioactive void filler inserts, whereas the porous cages integrate with the bone along the surfaces. The difference in the process of fusion can be explained by different cage structure and composition.

Ceramic Al_2_O_3_ cage is a synthetic porous tissue scaffold itself [12, 13]. The artificial material of the PEEK cage has no biological activity. There are multiple studies showing that stand-alone PEEK cages have a higher rate of pseudarthrosis [22, 23]. According to Ahmed et al., packed PEEK cages with fusion-promoting materials are preferable to empty PEEK cages because they have higher fusion and lower subsidence rates. In their review, empty cages had subsidence rates of 0–48.3% and fusion rates of 81.3–100% [24]. In general, the use of bone growth-stimulating agents leads to better results in fusion [17]. Our PEEK cage has two chambers that we fill with a CaHPO_4_-collagen composite, which has excellent osteoinductive properties. [25, 26]. In addition, two small metal wedges in the middle of the PEEK cage, which serve for better anchoring into the endplates, may hypothetically have another benefit—improving the migration of bone and blood cells from the small perforation of vertebral body endplates. The fusion rate in the PEEK group was higher at all FU time intervals. We assume that the inclusion of the biologics improved fusion in this group. The price of both implants was almost the same; however, the CaHPO_4_-collagen composite filler increased the price by an additional 25%. We are not aware of any side effects associated with the use of this composite.

We separately assessed the achievement of grade I fusion, which is complete bilateral fusion from a radiological point of view. However, without knowing the correlation with clinical outcomes, we cannot assess the clinical significance of this complete fusion achievement.

Peri-implant osteolysis was more common in the PEEK group. There is no literature evidence of osteolysis around the Al_2_O_3_ cage; on the contrary, PEEK wear particles caused significant peri-implant osteolysis [27, 28]. The incidence of subsidence in the PEEK group is within the published range of 14.9–66% [10, 19–21, 29]; in contrast, the Al_2_O_3_ group showed incidence of subsidence below this range.

There may be some possible limitations to this study. The first, method of randomization, did lead to the formation of unequally sized groups. Allocating more patients (with a 2:1 ratio) to the group with an Al_2_O_3_ cage, which itself was the focus of the experiment, we wanted our results on this cage to be more accurate and precise. The second, the study focussed on the radiological evaluation of the fusion rate without evaluating its impact on the clinical outcome. The next limitation concerns the use of a bone growth-stimulating agent only in PEEK cages. CaHPO4-collagen composite sponge is a ready-to-use product of the same company as the PEEK cage that we have used. There is no possibility of combining this composite sponge with a porous and quite solid Al_2_O_3_ cage. Hypothetically, the same composite material, but in paste form, could be used in combination with an Al_2_O_3_ cage. Keppler et al. presented in 2020 a new paste-like bone-filling biomaterial based on a polysaccharide matrix, calcium phosphate, and aluminium oxide granulates. Good biocompatibility under in vitro and in vivo conditions was confirmed, such as the initiation of osseous ingrowth into the bone defect [30].

## Conclusions

The findings of this prospective comparative randomized study showed that porous Al_2_O_3_ cages do not lead to faster fusion initialization, a higher quality of fusion, or a better fusion rate at final FU compared to PEEK cages. The current study also showed that the fusion rate of Al_2_O_3_ cages is within the range of published results for various cages used in ACDF procedures. Moreover, Al_2_O_3_ cages presented a lower incidence of subsidence compared to published data. Consequently, we consider the porous Al_2_O_3_ cage as safe to use for a stand-alone cervical disc replacement.

## Data Availability

Datasets generated during this study are available from the corresponding author on reasonable request.

## Abbreviations

ACDF: Anterior cervical discectomy and fusion
PEEK: Polyetheretherketone
FU: Follow-up
CT: Computed tomography
